# Functional Analysis of Keto-Acid Reductoisomerase ILVC in the Entomopathogenic Fungus *Metarhizium robertsii*

**DOI:** 10.3390/jof7090737

**Published:** 2021-09-08

**Authors:** Yulong Wang, Shihong Liu, Xuebing Yin, Deshui Yu, Xiangyun Xie, Bo Huang

**Affiliations:** 1 Anhui Provincial Key Laboratory of Microbial Pest Control, Anhui Agricultural University, Hefei 230036, China; wyl2019@ahau.edu.cn (Y.W.); lsh1491416840@163.com (S.L.); yxb@stu.ahau.edu.cn (X.Y.); DeshuiYu@yeah.net (D.Y.); yuriname@163.com (X.X.); 2 Engineering Research Center of Fungal Biotechnology, Ministry of Education, Anhui Agricultural University, Hefei 230036, China

**Keywords:** *Metarhizium robertsii*, ILVC, reductase activity, transcriptomics analysis, mycelial growth, conidial germination

## Abstract

Ketol-acid reductoisomerase (ILVC) is the second enzyme in the branched-chain amino acid (BCAA) biosynthesis, which regulates many physiological activities in a variety of organisms from bacteria to fungi and plants. In this work, function mechanisms of ILVC in *Metarhizium robertsii* Metchnikoff (Hypocreales: Clavicipitaceae) were explored with site-directed mutagenesis, reductase activity assays and transcriptomics analysis. The reductase activity assays showed that ILVC from phytopathogenic fungi exhibited significantly higher activities than those from entomopathogenic fungi but lower than those from yeast. Site-directed mutagenesis and enzymatic activities of MrILVC with different active-site mutants (Arg-113, Ser-118, Asp-152, Asp-260, and Glu-264) confirmed that active sites of MrILVC are conserved with plant and bacterial ILVCs. Deleting *MrilvC* causes the complete failures of vegetative growth and conidial germination, feeding with branched-chain amino acids (BCAAs) recovers the fungal growth but not conidial germination, while both characteristics are restored when supplemented with yeast extract. Compared to Δ*MrilvC* cultured in czapek agar (CZA), plenty of genes involved in the biosynthesis of antibiotics and amino acids were up- or down-regulated in the wild type or Δ*MrilvC* feeding with either BCAAs or yeast extract. Further analysis showed some genes, such as *catalase A*, participate in mycelial growth and conidial germination was down-regulated in Δ*MrilvC* from CZA, revealing that MrILVC might control the fungal development by gene regulation and BCAAs or yeast extract could play partial roles of MrILVC. This study will advance our understanding of ILVC function mechanisms in fungi.

## 1. Introduction

Valine, leucine, and isoleucine form the small group of branched chain amino acids (BCAAs), which are synthesized in bacteria, plants, and fungi, but not in animals [1,2]. The biosynthesis of these amino acids shows high similarity between organisms of the different kingdoms of life, of which valine and isoleucine synthesis are carried out by the same enzymes, and leucine is created from α-ketoisovalerate (a transamination precursor of valine) [3]. However, striking differences on the capabilities to synthesize or degrade branched-chain amino acids exist, and BCAAs in different groups of organisms also have some unique functions and features, revealing the various function mechanisms of BCAAs among different organisms [4].

The conservation in fungi but absence in mammals of the BCAA biosynthetic pathway makes it as the target for herbicides, fungicides, and antimicrobial compounds [5,6]. Acetohydroxyacid synthase (AHAS), the first enzyme in this pathway, has been targeted and inhibited by several commercial herbicides, such as sulfonanilides, imidazolinones, and sulfonylureas [7,8]. Ketol-acid reductoisomerase (ILVC, KARI, or ILV5), downstream of AHAS in the BCAA biosynthetic pathway, catalyzes the conversion of 2-acetolactate and 2-aceto-2-hydroxybutyrate to 2,3-dihydroxyisoverate and 2,3-dihydroxy-3-methylvalerate, respectively. Although there are no commercial herbicides targeting ILVC, some compounds have been reported as potent competitive inhibitors of ILVC in vitro, such as HOE 704, IpOHA, and CPD [6]. To date, BCAA enzyme inhibitors have shown antimicrobial effects on some harmful pathogenic microorganisms, including *Fusarium graminearum* Petch (Hypocreales: Nectriaceae), *Pseudomonas aeruginosa* Migula (Pseudomonadales: Pseudomonadaceae) and *Candida albicans* Berkhout (Saccharomycetales: Saccharomycetaceae), but whether they have inhibitory effects on pest pathogenic fungi are still uncharted [6,9,10].

ILVC is a bifunctional enzyme that catalyzes two quite different reactions, but occur at a common active site, acting both as an isomerase and as a reductase [11]. ILVCs have a GxGxx(G/A)xxx(G/A) motif as a binding site for NADP(H), and Mg^2+^ is required for binding NADP(H) [9,12,13]. Site-directed mutagenesis and activity assays showed that plant and bacterial ILVCs have evolved different mechanisms of induced fit to prepare the active site for catalysis [9,12]. Previous sequence comparison revealed that those active residues are highly conserved across plant, fungal, and bacterial ILVCs, but the gene or genes responsible for ILVC activity in fungi have not been investigated in any detail. In addition, functional analysis of the ILVC has been carried out in bacteria, plants, and fungi; however, transcriptome analyses for the blocking of BCAA biosynthesis remain unclear [6,14].

In our previous study, we found that ILVC is associated with conidial germination and fungal pathogenicity in the insect–pathogenic fungus *Metarhizium robertsii* Metchnikoff (Hypocreales: Clavicipitaceae), formerly classified as *Metarhizium anisopliae*, and the *ilvC*-deleted mutant failed to germinate on CZA complemented with three BCAAs. Complementation of BCAAs cannot recover the conidial germination for the *ilvC*-deficient strain [14]. This study seeks to further characterize *ilvC* in *M. robertsii* by phenotypic and comparative analyses of its deletion mutants. Relative catalytic activities of *ilvC* mutants in five active-site residues were assayed and catalytic activities of ILVCs from different fungi were compared.

## 2. Materials and Methods

### 2.1. Phylogenetic Analysis

BLAST searches were carried out to identify different eukaryotic ortholog of the *Saccharomyces cerevisiae* (GenBank accession number AJV63457.1) and *Arabidopsis thaliana* (NP_001078309.1) ILVC protein. ILVC sequences were aligned using ClustalX, and then a neighbor-joining (NJ) tree was generated using MEGA 7.0 software. ILVC sequences from *Streptococcus pneumoniae* (WP_000218054.1), *Slackia exigua* (WP_040619008.1), *Alicyclobacillus acidocaldarius* (WP_008340213.1), *Mycobacterium tuberculosis* (MXI72316.1), *Pseudomonas aeruginosa* (WP_070331278.1), *Escherichia coli* (MQL15051.1), *A. thaliana*, *Spinacia oleracea* (1QMG_A), *Oryza sativa* (EEE64772.1), *M. robertsii* (XP_007819198.1), *F. graminearum* (XP_011319056.1), *Aspergillus fumigatus* (XP_754177.1), and *S. cerevisiae* were used for phylogenetic analysis.

### 2.2. Fungal Strain and Maintenance

The *ilvC*-deficient (Δ*MrilvC*), and complemented strains (Comp) were constructed from the wild-type (WT) *M. robertsii* strain ARSEF 23 (ATCC no. MYA-3075) in our previous report [14]. All strains grown on SDAY (4% glucose, 1% peptone, 1% yeast extract powder, and 1.5% agar, *w*/*v*) at 28 °C in the dark. Conidia were harvested in a 0.05% Tween 80 aqueous solution from different strains cultured with 20 days and filtered through sterile nonwoven fabric for mycelia removal. The conidial concentration was determined using a hemocytometer and diluted to 1 × 10^7^ conidia mL^−1^.

### 2.3. Site-Directed Mutagenesis, Protein Expression and Purification

Site-directed mutagenesis was performed with TaKaRa MutanBest kit (TaKaRa, Beijing, China). Briefly, the *ilvC* sequence was amplified by PCR from the cDNA template and PCR products were inserted into pOT2 plasmid. pOT2-Ilvc plasmid was amplified with designed mutant primers by PCR, and blunted using Blunting Kination Enzyme Mix, then ligated using ligation solution I in the kit. Plasmids were transformed into *Escherichia coli* Migula (Enterobacterales: Enterobacteriaceas) DH5α (TransGen, Beijing, China) and verified by DNA sequencing. Five amino acid residues contacting both NADP(H) and Mg^2+^ are conserved among bacteria, fungi, and plants; thus, their 5 active-site residue mutageneses were performed as mentioned above, respectively [15].

The recombinant ILVC protein was expressed in *E. coli* [16]. Briefly, *ilvC* with a 6× His-tag sequence at the C-terminus was amplified by PCR from the cDNA template or pOT2-Ilvc, and PCR products were inserted into pET-28b (+) vector (Novagen, Beijing, China), then transformed into *E. coli* strain BL21 (DE3)-competent cells (TransGen, Beijing, China). ILVC protein expression was induced by the addition of isopropyl β-D-thiogalactoside (IPTG) to a final concentration of 0.5 mM and purified with Ni-NTA agarose (Qiagen, Chatsworth, CA, USA) using a nickel-ion affinity column (Qiagen). Protein purity was monitored by SDS-PAGE.

### 2.4. Reductase Activity Assays

The enzymatic activity assays for ILVC protein were performed [15,17,18]. Firstly, ILVC protein (1 μg) was pre-incubated in the activity assay buffer (50 mM Tris-HCl pH 7.0, 1 mmol DTT, 200 mmol NADPH, 10 mmol MgCl_2_) at room temperature for 30 min. Secondly, 2-Acetolactate was added to the reaction mixture. The enzymatic activity was determined by monitoring the rate of NADPH oxidation at 340 nm. Six independent experiments were repeated. In this study, reductase activity of ILVC from *S. cerevisiae*, *A. fumigatus*, *F. graminearum*, *Beauveria bassiana* (Hypocreales: Cordycipitaceae) and *M. robertsii* were determined by protein expression and purification from *E. coli*.

### 2.5. The Sensitivity to Herbicides

Mycelial growth tests were performed on SDAY plates supplemented with the following AHAS inhibitors at the concentrations of 10 μg mL^−1^: tribenuron-methyl, rimsulfuron, metsulfuron-methyl, halosulfuron-methyl, chlorimuronethyl, imazethapyr and imazapyr (Henan Tianfu Chemical Co., Ltd., Henan, China). The percentage of inhibition of mycelial radial growth was calculated using the formula: mycelial growth inhibition = [(*C* − *N*)/(*C* − 5)] × 100, where *C* is colony diameter of the control, and *N* is that of the treatment with AHAS inhibitors [6]. Each experiment was carried out with six replicates.

### 2.6. Quantitative RT-PCR Analysis of MrilvC

100-μL aliquots of a conidial suspension collected from strains cultured on SDAY were spread on SDAY at 28 °C in the dark; at 18 h, 48 h, and 96 h, cultured strains were collected. Conidial suspensions were put on cicada (*Cryptotympana atrata* Westwood (Hemiptera: Cicadidae)) hind wings [19]. For the samples of *M. robertsii* during the infection progress, conidia were used for cuticle infection by dipping the fifth-instar *Galleria mellonella* L. (Lepidoptera: Pyralidae) larvae into a conidial suspension (1 × 10^7^ conidia mL^−1^) for 10 s. Then, the infected larvae were maintained in a stable photoperiod with 16 h of light and 8 h of dark at 28 °C without feeding. Infected *G. mellonella* were collected at 24 h, 48 h, and 96 h. Quantitative RT-PCR was performed [20]. In brief, TRIzol reagent (Invitrogen, Carlsbad, CA, USA) was used for RNA extraction, and complementary DNA were synthesized with a gDNA remover kit (Toyobo, Osaka, Japan) as the manufacturer’s instructions. *Glyceraldehyde 3-phosphate dehydrogenase* (GAPDH) was used as the control. Gene amplification was achieved using the Bio-Rad CFX96 real-time PCR detection system (Bio-Rad, Hercules, CA, USA). Transcripts of *MrilvC* were normalized to the control gene, and the 2^−ΔΔCt^ method was used to calculate the relative expression level (mean ± standard deviation) of each gene. Three independent experiments were performed for each gene.

### 2.7. Phenotypic Assays

A 4-mm-diameter agar plug of each strain from the border of a 5-day-old colonies on SDAY plates was centrally attached onto CZA (0.3% NaNO_3_, 0.1% K_2_HPO_4_, 0.05% MgSO_4_·7H_2_O, 0.05% KCl, 0.001% FeSO_4_·7H_2_O, 3% sucrose, and 1.5% agar, *w*/*v*), CZA + AA (CZA with 1 mM Ile, Leu, and Val) or CZA + yeast (CZA with 1% yeast extract powder, *w*/*v*) at 28 °C under a 12 h:12 h light/dark cycle. The radial growth (colony diameter) of the vegetative mycelia was measured daily from the back sides of the plates with vernier caliper. For the assessment of different strain conidial yield capacity, 100-μL aliquots of a conidial suspension were spread on CZA, CZA + AA, and CZA + yeast. From day 3 onwards, 5-mm diameter colony disks were excised at random from the plates daily and washed off into 2 mL of 0.05%. The concentration of the conidial suspension was determined by a hemocytometer under a microscope.

### 2.8. Transcriptomics Analysis

A 4-mm-diameter agar plug of each strain from the border of a 5-day-old colony containing both mycelium and conidium on SDAY plates was centrally attached onto CZA, CZA + AA, and CZA + yeast plates for 24 h when both mycelial growth and conidial germination happened. RNA samples, library construction, and sequencing were performed [21]. Total RNA was extracted and treated with recombinant DNase I (rDNase I; Sigma, St Louis, MO, USA) to remove any potential genomic DNA contamination. To maximize target coverage, equal amounts of total RNA from the three replicates of strains were pooled as a sample library for RNA-Seq library construction, and then a total of 5 µg of total RNA were prepared for RNA-Seq.

Total RNA was purified with oligo(dT) beads and fragmented into small pieces, treated with RNase H for RNA removement after the complementary DNA synthesized. The cDNA library was constructed and sequenced using the Illumina Hi-Seq 4000 platform with a single-end (single reads of 50 bases) sequencing strategy at Beijing Genomics Institute (BGI, Shenzhen, China). Clean reads were obtained by removing the raw reads, then used to map the *M. robertsii* genome (https://www.ncbi.nlm.nih.gov/genome/?term=Metarhizium+robertsii (accessed on 31 January 2020)). The gene expression level was normalized using the fragments per kilobase per million mapped reads (FPKM) method [22]. Rigorous algorithms were applied to identify differential expressed genes (DEGs) with a false discovery rate (FDR) ≤ 0.001 and an absolute value of the log_2_ ratio ≥ 1 as thresholds [23]. Gene ontology (GO) functional analysis (Blast2GO, https://www.blast2go.org/ (accessed on 13 March 2020)) and Kyoto Encyclopedia of Genes and Genome (KEGG) pathway enrichment analysis was performed to further understand the biological functions of the identified DEGs. In addition, a total of 20 genes involved in conidial germination or mycelial growth in this study were selected to perform quantitative RT-PCR validation. All primers used in this study were listed in Table S1.

### 2.9. Statistical Analysis

Data were analyzed by one-way analysis of variance, followed by Dunnett’s multiple comparison and Tukey’s test using the SPSS v23.0 software. All results were expressed as the mean ± standard error of the mean (SD). *p* < 0.05 was considered statistically significant.

## 3. Results

### 3.1. Phylogenetic Analysis of ILVC from Eukaryotes

**Figure 1:**
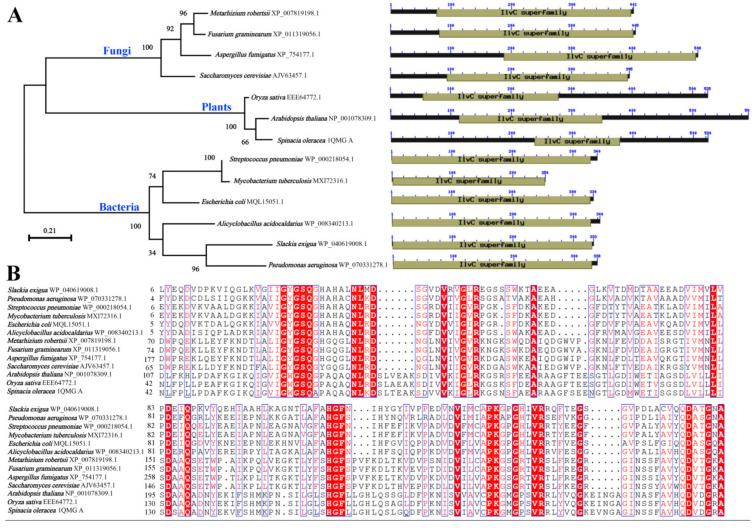
ILVCs in diverse organisms. (**A**) Phylogenetic analysis of ILVCs. (**B**) Conserved domain arrangements of the ILVCs.

Phylogenetic analysis showed that ILVC protein from fungi, plants, and bacteria were clustered in respective clades. Analysis of the domain architecture showed that ILVC protein from plants had the shortest conserved domain (ILVC superfamily) and bacterial ILVC sequences were the longest in length among the eukaryotes (Figure 1A).

Five amino acid residues contacting both NADP(H) and Mg^2+^ are also conserved in fungi, bacteria, and plants, indicating that all these ILVCs have either reductase or isomerase activity [11]. In *M. robertsii*, residues contacting both NADP(H) and Mg^2+^ are Arg-113, Ser-118, Asp-152, Asp-260, and Glu-264 (Figure 1B).

### 3.2. Enzymatic Activity of ILVC from Different Fungi and MrILVC with Active-Site Mutant

**Figure 2:**
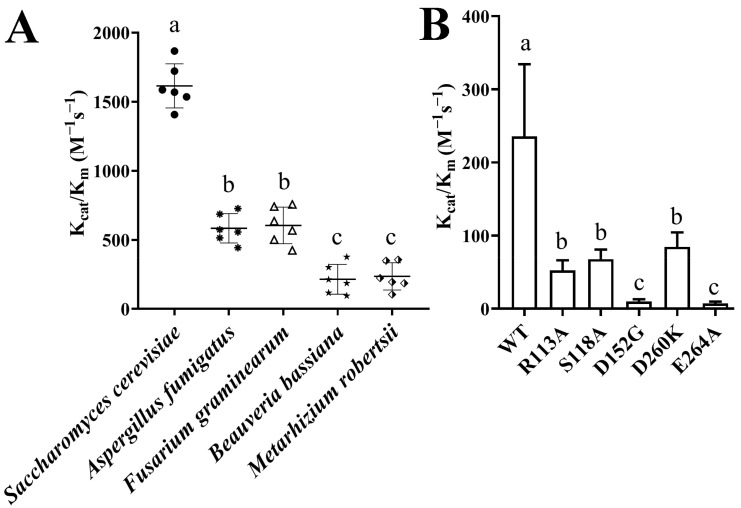
Kinetic parameters of ILVCs. (**A**) Kinetic parameters of different fungal ILVCs. (**B**) Kinetic parameters of MrILVC and its mutants. Different lowercase letters marked on the bars in each graph denote significant differences (*p* < 0.05). Error bars, standard deviation from six replicate assays.

As shown in Figure 2A, ILVC from phytopathogenic fungi (*A. fumigatus* and *F. graminearum*) exhibited significantly higher activities than those from entomopathogenic fungi (*B. bassiana* and *M. robertsii*) but lower than that from *S. cerevisiae*.

All the mutants (R113A, S118A, D152G, D260K, and E264A) exhibited significantly reduced activities compared to those of WT (Figure 2B), confirming that the roles of those residues on ILVC activities were conserved among different fungi [15].

### 3.3. Deletion of MrILVC Increased Tolerance to AHAS Inhibitors

**Figure 3:**
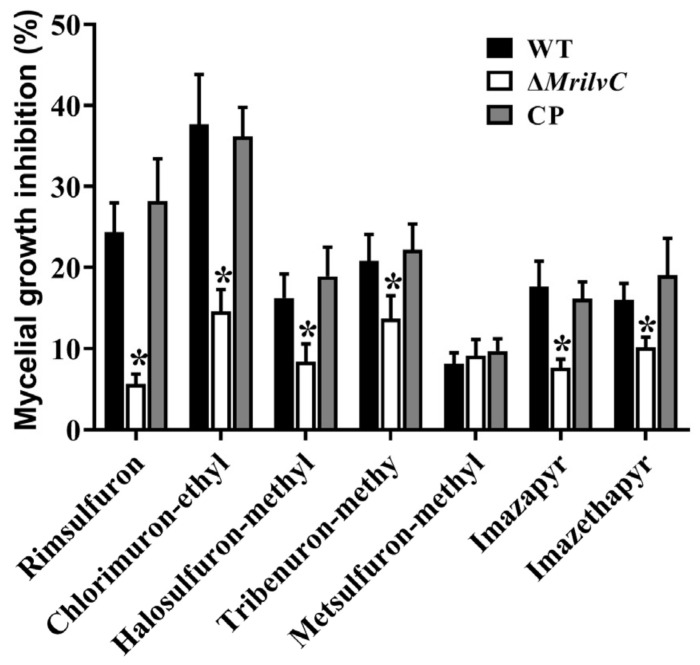
The MrILVC deletion mutant is resistant to AHAS inhibitors. Error bars represent the SEM of six replicate assays. * Significantly different expression with respect to the WT standard (*p* < 0.05).

All AHAS inhibitors showed significant inhibitory effects with 10~40% mycelial growth inhibition on the wild-type (Figure 3). Compared with the WT, Δ*MrilvC* showed significant resistance to tribenuron-methyl, rimsulfuron, halosulfuron-methyl, chlorimuronethyl, imazethapyr, and imazapyr, but not metsulfuron-methyl, suggesting that those six AHAS inhibitors might inhibit the MrILVC activity.

### 3.4. ILVC Contributes to Mycelial Growth and Conidial Germination

**Figure 4:**
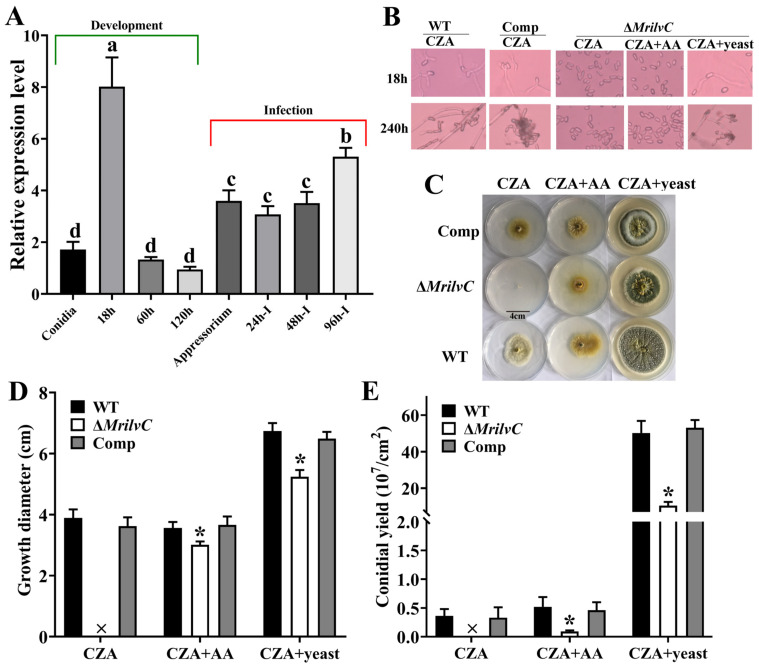
Deletion of MrILVC affected conidial germination, hyphal growth, and conidiation. (**A**) Transcriptional profiling of *MrilvC* at different developmental and infection stages. (**B**) Microscopy observation of WT and Δ*MrilvC* in different media. (**C**) Colony morphologies of WT and Δ*MrilvC* in different media. (**D**,**E**) Growth diameters and conidial yields of different strains cultured on CZA, CZA + AA, and CZA + yeast. Error bars represent the SEM of six replicate assays. * Significantly different expression (*p* < 0.05). Different lowercase letters marked on the bars in each graph denote significant differences (*p* < 0.05).

*MrilvC* expression sharply increased expression at the stage of conidial germination and decreased rapidly from 60 h (Figure 4A). Additionally, *MrilvC* expression, increased rapidly after infection with conidia, was highly expressed from 96 h-I when *M. robertsii* has entered into the hemocoel.

Conidia of the Δ*MrilvC* mutant were unable to germinate on CZA medium supplied with all three BCAAs (Ile, Leu and Val), but were able to germinate on CZA medium supplemented with yeast extract (Figure 4B). The *MrilvC*-deficient strain was unable to grow on CZA plates, but could grow on CZA medium supplemented with BCAAs or yeast extract despite the colony being smaller than that of the WT (Figure 4C,D). In addition, in contrast to the WT and complemented transformant, the *MrilvC*-deficient strain showed a dramatic decrease in the conidial yields in CZA medium supplemented with BCAAs or yeast extract (Figure 4E).

### 3.5. RNA-Seq Analysis of ΔMrilvC on Different Medium

**Figure 5:**
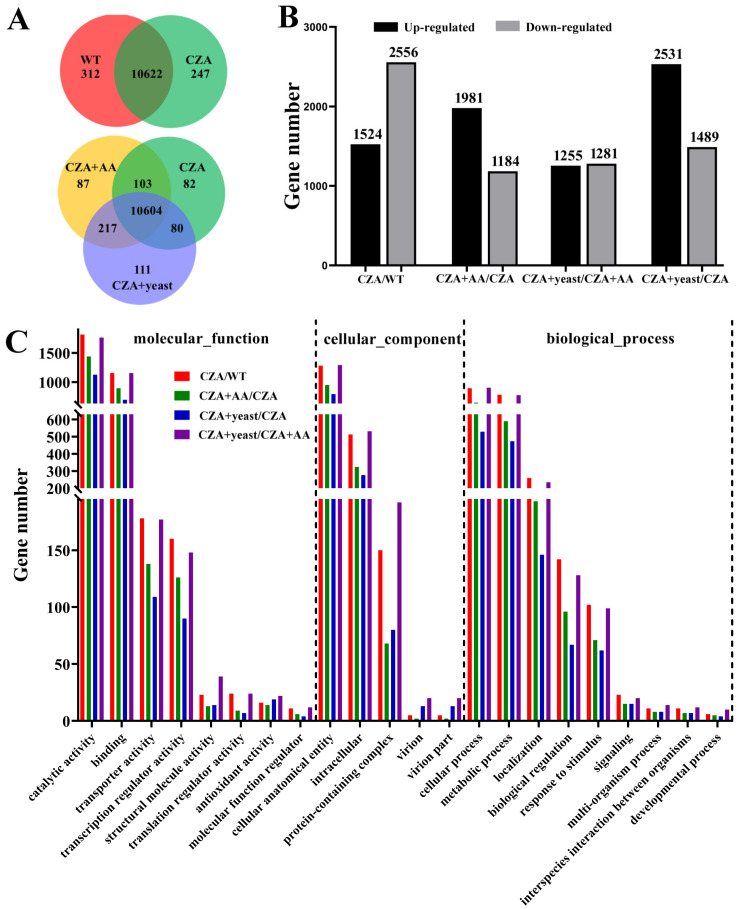
Gene expression in the wild-type strain cultured on czapek agar (WT) and Δ*MrilvC* cultured on czapek agar (CZA) supplemented with BCAAs (CZA + AA) or yeast extract (CZA + yeast). (**A**) Venn diagram showing the distribution of expressed genes. (**B**) DEGs from WT or CZA or CZA + AA or CZA + yeast samples. (**C**) GO enrichment analyses of the DEGs from different samples. WT: The WT strain cultured on CZA plates; CZA: Δ*MrilvC* mutant grown on CZA plates; CZA + AA: Δ*MrilvC* mutant grown on CZA + AA plates; CZA + yeast: Δ*MrilvC* mutant grown on CZA + yeast plates.

Totals of 10,934 (WT) and 10,869 (CZA) genes were detected in the WT and Δ*MrilvC* mutant grown on CZA plates; 11,011 (CZA + AA) and 11,012 (CZA + yeast) genes expressed in the Δ*MrilvC* mutant grow on CZA + AA and CZA + yeast plates (Figure 5A). Of these genes, 312 and 247 genes were found to be specifically expressed in WT and CZA, respectively, indicating the potential roles of MrILVC in their expression. In addition, 82, 87, and 111 genes were expressed specifically in CZA, CZA + AA and CZA + yeast samples, respectively.

Compared with the WT, 1524 genes were significantly up-regulated while 2556 genes were significantly down-regulated after the deletion of *MrilvC* in *M. robertsii* grown on CZA plates (Figure 5B). It is worth mentioning that, compared with the WT, all genes encoding key enzymes of BCAA biosynthesis were significant up-regulated in the CZA, indicating that the disruption of BCAA metabolism contributes to the expression levels of those key enzymes involved in BCAA biosynthesis (Figure S1). Moreover, a total of 3165 and 4020 genes were differentially expressed between CZA + AA/CZA + yeast and CZA samples, respectively, and 2536 DEGs were found between CZA + AA and CZA + yeast samples.

Based on sequence homology, the sequences significantly enriched 22 functional groups (Figure 5C). Catalytic activity, binding, cellular anatomical entity, intracellular and cellular process, and metabolic process were the most commonly annotated terms in each of the three GO term categories. The DEGs of WT/CZA were enriched in 17 KEGG pathways (*p* < 0.05) (Table 1). Of these pathways, biosynthesis of antibiotics and biosynthesis of amino acids were the most highly enriched KEGG pathways, indicating that the severity affection of normal biosynthesis in *M. robertsii* is caused by the MrILVC deletion. The biosynthesis of antibiotics and biosynthesis of amino acids were also the most enriched pathways in CZA + AA/CZA and CZA + yeast/CZA, but not in CZA + yeast/CZA + AA. As mentioned above, plenty of DEGs, the expression levels of these genes were validated using quantitative RT-PCR, from WT/CZA (116), CZA + AA/CZA (95), and CZA + yeast/CZA (106) were enriched in the biosynthesis of amino acids. In addition to DEGs involved in BCAA biosynthesis, some DEGs participated in the biosynthesis of other amino acids, such as lysine, proline, and arginine (Table S2).

Furthermore, DEGs from CZA + yeast/CZA + AA were mostly involved in amino sugar and nucleotide sugar metabolism (109), followed by those involved in glycine, serine, and threonine metabolism (58), tryptophan metabolism (48), and phenylalanine metabolism (44).

### 3.6. MrILVC Affects Expression of Genes Involved in Mycelial Growth and Conidial Germination

**Figure 6:**
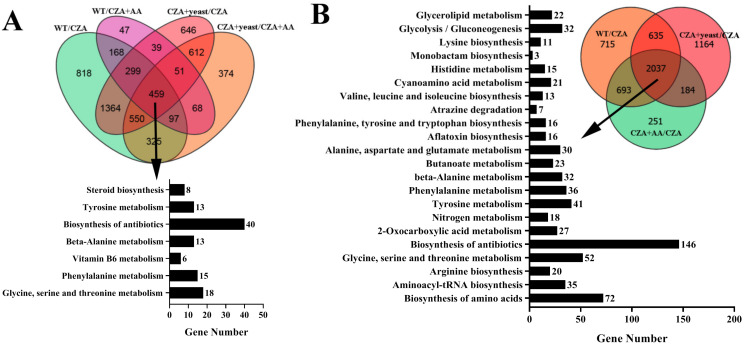
Venn diagrams of DEGs between conidial germinated and non-germinated samples (**A**) and mycelial growth and non-growth samples (**B**). DEGs in common were analyzed with KEGG enrichment. WT: The WT strain cultured on CZA plates; CZA: Δ*MrilvC* mutant grown on CZA plates; CZA + AA: Δ*MrilvC* mutant grown on CZA + AA plates; CZA + yeast: Δ*MrilvC* mutant grown on CZA + yeast plates.

Venn analysis showed that a total of 459 DEGs were found between conidial germinated and inhibited samples (Figure 6A and Table S3). Among of them, more than 100 DEGs were significantly enriched in seven function classes with function classification, for which biosynthesis of antibiotics (40 DEGs) was the most highly enriched pathway. Based on the expression patterns, DEGs with higher expression levels in conidial germinated than inhibited samples were listed in Table 2. Among of them, *catalase A* and *SAM-dependent methyltransferase* genes played important roles in the fungal conidial germination [24,25].

Venn analysis of DEGs showed 2037 DEGs co-existed in WT/CZA, CZA + AA/CZA, and CZA + yeast/CZA, and were significantly enriched in 22 function classes (Figure 6B and Table S4). DEGs with higher expression levels in mycelial growth than inhibited samples were listed in Table 3. Some genes involved in mycelial growth, such as *Alcohol dehydrogenase*, *catalase A* and *chitinase* were lower expressed in CZA than other samples [26,27,28].

In addition, 20 genes involved in conidial germination or mycelial growth were shown to be up-regulated in WT/CZA, CZA + AA/CZA, and CZA + yeast/CZA from RNA-Seq data. Quantitative RT-PCR analysis revealed 19, 18, and 18 genes were significantly up-regulated in WT/CZA, CZA + AA/CZA, and CZA + yeast/CZA, respectively, supporting the validity of our transcriptomics results (Figure S2).

## 4. Discussion

ILVCs can be separated into two classes based on the length of their polypeptides: class I proteins with ~340 amino acid residues, and class II proteins with more than 520 residues [12,13]. In our study, ILVCs in fungi had various lengths from 400 to 500 residues, which were located between the plant and the bacteria in length. Domain architecture analysis showed that all ILVCs have the IlvC domain, and plant ILVCs have a C-terminal extension, play dual roles in forming the active site, but are missing in ILVCs of both bacteria and fungi, confirming that ILVCs from bacteria and fungi belong to the class I group and suggesting they may have similar function mechanisms which were different from plant ILVCs [13]. Moreover, multiple-sequence alignment of ILVCs showed that residues constituting NADP(H) and Mg^2+^ binding sites are well conserved, while the overall length of each KARI protein is different.

ILVCs from different organisms have evolved different mechanisms of induced fit to prepare the active site for catalysis [12]. Enzymatic activity assays showed that ILVC from phytopathogenic fungi exhibited significantly higher activities than those from entomopathogenic fungi but lower than that from *S. cerevisiae*, suggesting that there is a direct link between the ILVC catalysis activity and the characteristics of fungal life cycles. ILVC and its orthologous proteins have either reductase or isomerase activity, but only the reductase activity was explored in this study because of the difficulty and reaction complex of isomerase action [11,15]. Structure-based multiple sequence alignment of ILVCs from bacteria and plants revealed that five amino acid residues contacting both NADP(H) and Mg^2+^ are conserved [15]. In our study, sequence alignment and enzymatic activities of MrILVC with different active-site mutants confirmed that those residues are also conserved in fungal ILVCs.

To date, BCAA enzyme inhibitors shown antimicrobial effects on some harmful pathogenic microorganisms, such as *F. graminearum*, *P. aeruginosa,* and *C. albicans* [6,9,10]. In this study, all tested AHAS inhibitors showed significant inhibitory effects with 10~40% mycelial growth inhibition on *M. robertsii*, revealing that the use of entomopathogenic fungi to control pests should be avoided during the AHAS inhibitors’ used area. Consistent with the observation in *F. graminearum*, deleting *ilvC* showed significant resistance to tribenuron-methyl, rimsulfuron, halosulfuron-methyl, chlorimuronethyl, imazethapyr and imazapyr, suggesting that those six AHAS inhibitors might inhibit the fungal ILVC activity [6].

In this study, qRT-PCR showed *MrilvC* expression sharply increased expression at the stage of conidial germination and infection, which is consistent with the impairment of the full virulence on insects treated with direct injection or cuticle infection in our previous study [14]. BCAAs account for more than 20% of total protein amino acids and are an important constituent of the amino acid pool in organisms [29]. Transcriptomics analysis showed lots of genes involved in both metabolism and biosynthesis of amino acids were differentially expressed after the *MrilvC* deletion, which implied that the blocking of BCAAs impaired protein functioning in those pathways.

Catalase A, a monofunctional catalase in fungi, played crucial roles in various aspects of cell physiology and cellular differentiation, including conidial germination and mycelial growth [25,30]. In *M. robertsii*, the expression of *catalase A* was down-regulated after the deletion of *MrilvC*, while it returned to an expression level similar to the WT in Δ*MrilvC* feeding with yeast extract. In addition, some genes involved in conidial germination or mycelial growth, such as *Alcohol dehydrogenase*, *SAM-dependent methyltransferase,* and *chitinase*, showed similar expression patterns with *catalase A*. From these results, *MrilvC* played critical roles in fungal development by regulating those gene expressions, and characteristics of Δ*MrilvC* could be partially restored to the WT with gene regulation by feeding with BCAAs or yeast extract. It is worth mentioning that deleting *MrilvC* led to the noticeable increase in the expression of genes involved in BCAA biosynthesis, and we speculated that BCAA levels could affect its biosynthesis and the disruption of BCAA biosynthesis by deleting *MrilvC,* resulting in those genes being significant up-regulated; more experiments are needed to explore those patterns.

In conclusion, our data indicate that MrILVC plays critical roles in controlling conidial germination and mycelial growth by the regulation of metabolism and biosynthesis of amino acids. ILVCs from different fungi revealed distinct reductase activity. Active sites of MrILVC are conserved with plant and bacterial ILVCs; one mutation among those exhibited significantly reduced activities. Data from this study advance our understanding of the function of ILVCs in entomopathogenic fungi, which would contribute to future BCAA metabolism investigations.

## Figures and Tables

**Table 1:** KEGG analysis of differentially expressed genes.

Pathway ID	Pathway Name	Gene Number			
		CZA/WT	CZA + AA/CZA	CZA + Yeast/CZA	CZA + Yeast/CZA + AA
ko01230	Biosynthesis of amino acids	116	95	106	-
ko00260	Glycine, serine, and threonine metabolism	91	76	82	54
ko01130	Biosynthesis of antibiotics	267	221	259	-
ko00970	Aminoacyl-tRNA biosynthesis	51	39	47	-
ko01210	2-Oxocarboxylic acid metabolism	45	35	-	-
ko00220	Arginine biosynthesis	26	22	22	-
ko00360	Phenylalanine metabolism	62	60	56	44
ko00910	Nitrogen metabolism	28	24	26	-
ko00410	beta-Alanine metabolism	54	44	-	-
ko00650	Butanoate metabolism	36	29	-	-
ko00630	Glyoxylate and dicarboxylate metabolism	43	-	-	-
ko00920	Sulfur metabolism	24	-	51	-
ko00310	Lysine degradation	47	-	40	-
ko00350	Tyrosine metabolism	63	55		-
ko00740	Riboflavin metabolism	25	-	27	-
ko00750	Vitamin B6 metabolism	13	-	-	-
ko00290	Valine, leucine, and isoleucine biosynthesis	19	17	-	-
ko00380	Tryptophan metabolism	-	63	67	48
ko00400	Phenylalanine, tyrosine, and tryptophan biosynthesis	-	22	23	-
ko00250	Alanine, aspartate, and glutamate metabolism	-	41	46	-
ko00340	Histidine metabolism	-	21	-	-
ko00254	Aflatoxin biosynthesis	-	21	-	-
ko00603	Glycosphingolipid biosynthesis—globo and isoglobo series	-	6	-	-
ko02010	ABC transporters	-	28	-	-
ko00561	Glycerolipid metabolism	-	31	37	-
ko00052	Galactose metabolism	-	22	-	-
ko00330	Arginine and proline metabolism	-	41	-	-
ko00460	Cyanoamino acid metabolism	-	27	36	-
ko00300	Lysine biosynthesis	-	14	-	-
ko00520	Amino sugar and nucleotide sugar metabolism	-	-	-	109
ko00072	Synthesis and degradation of ketone bodies	-	-	11	-
ko00450	Selenocompound metabolism	-	-	12	-
ko01200	Carbon metabolism	-	-	96	-
ko00240	Pyrimidine metabolism	-	-	31	-

WT: The WT strain cultured on CZA plates; CZA: Δ*MrilvC* mutant grown on CZA plates; CZA + AA: Δ*MrilvC* mutant grown on CZA + AA plates; CZA + yeast: Δ*MrilvC* mutant grown on CZA + yeast plates.

**Table 2:** Potential genes involved in conidial germination.

Gene Name	FPKM			
	WT	CZA + Yeast	CZA + AA	CZA
hypothetical protein	214.29	706.56	103.95	20.36
catalase A	99.73	59.86	19.71	4.41
DNA-binding WRKY domain-containing protein	46.66	55.6	21.36	5.12
bacterial-type extracellular deoxyribonuclease	55.33	21.65	8.84	3.24
hypothetical protein MAA_10685	22.67	44.16	8.14	0.1
macrophomate synthase	32.71	31.71	6.15	0.08
Cytochrome P450 CYP684F1	30.19	11.43	1.82	0.16
NAD(P)-binding domain protein	8.44	30.33	3.3	0.17
hypothetical protein	6.03	31.47	2.19	0.33
lanosterol synthase	8.76	30.28	2.79	2.82
HypA	21.51	12.53	6.18	0.34
D-isomer specific 2-hydroxyacid dehydrogenase	15.77	8.56	0.82	0.24
methyltransferase	16.66	11.94	4.87	0.92
SAM-dependent methyltransferase	16.22	10.06	2.6	1.32
NAD(P)-binding domain protein	10.49	18.05	4.5	1.94
hypothetical protein	5.59	18.58	2.04	0.37
bacterial-type extracellular deoxyribonuclease	12.56	14.15	5.36	2.05
membrane copper amine oxidase	9.93	9.4	3.94	1.79
NADP-dependent alcohol dehydrogenase C	6.71	7.22	2.98	0
Cytochrome P450 CYP68N3	1.6	2.4	0.21	0

WT: The WT strain cultured on CZA plates; CZA: Δ*MrilvC* mutant grown on CZA plates; CZA + AA: Δ*MrilvC* mutant grown on CZA + AA plates; CZA + yeast: Δ*MrilvC* mutant grown on CZA + yeast plates.

**Table 3:** Potential genes involved in mycelial growth.

Gene Name	FPKM			
	WT	CZA + Yeast	CZA + AA	CZA
sarcosine oxidase	2856.28	3610.63	1698.84	765.77
tryptophan synthase beta subunit-like PLP-dependent enzyme	220.33	216.52	115.84	5.72
hypothetical protein	258	238.66	152.01	20.68
alcohol dehydrogenase superfamily, zinc-type	265.12	239.15	182.46	42.76
catalase A	99.73	59.86	19.71	4.41
macrophomate synthase	32.71	31.71	6.15	0.08
hypothetical protein	62.78	59.9	38.78	6.99
aldehyde dehydrogenase	103.97	90.51	74.29	30.37
Chitinase	72.67	31.01	15.75	0.3
bacterial-type extracellular deoxyribonuclease	55.33	21.65	8.84	3.24
glutamate decarboxylase	149.42	125.48	112.82	49.62
Amine oxidase	38.32	37.46	25.08	3.05
hypothetical protein	184.05	33.08	22.57	2.77
Cytochrome P450 CYP684F1	30.19	11.43	1.82	0.16
hypothetical protein	31.27	19.17	10.99	0
hypothetical protein	201.58	43.19	35.55	14
methyltransferase	16.66	11.94	4.87	0.92
aldose 1-epimerase	33.08	29.01	22.51	6.58
HypA	21.51	12.53	6.18	0.34
membrane copper amine oxidase	9.93	9.4	3.94	1.79
alpha/beta hydrolase fold-3	40.18	35.92	30.48	13.89
hypothetical protein	18.13	6.68	3.1	0.36
cytochrome P450 CYP5321A1	54.37	34.38	32.75	1.61
peroxisomal copper amine oxidase	2.92	2.07	1.96	0.9

WT: The WT strain cultured on CZA plates; CZA: Δ*MrilvC* mutant grown on CZA plates; CZA + AA: Δ*MrilvC* mutant grown on CZA + AA plates; CZA + yeast: Δ*MrilvC* mutant grown on CZA + yeast plates.

## Data Availability

Not applicable.

## Supplementary Materials

The following are available online at https://www.mdpi.com/article/10.3390/jof7090737/s1, Figure S1: Expression levels of genes involved in BCAA pathways from different samples, Figure S2: Quantitative RT-PCR validation of differential expressed genes identified in RNA-Seq analysis. WT: The WT strain cultured on CZA plates; CZA: ΔMrilvC mutant grow on CZA plates; CZA + AA: ΔMrilvC mutant grow on CZA + AA plates; CZA + yeast: ΔMrilvC mutant grow on CZA + yeast plates, Table S1: Primers used in this study, Table S2: Expression of genes involved in biosynthesis of amino acids, Table S3: Differential expressed genes between conidial germination and inhibited samples, Table S4: Differential expressed genes between mycelial growth and inhibited samples.
