# Supplementary figures and images for: Functional Analysis of Keto-Acid Reductoisomerase ILVC in the Entomopathogenic Fungus Metarhizium robertsii
Source: J Fungi (Basel). 2021 Sep 8;7(9):737. doi: 10.3390/jof7090737 (PMC8471054; doi:10.3390/jof7090737)

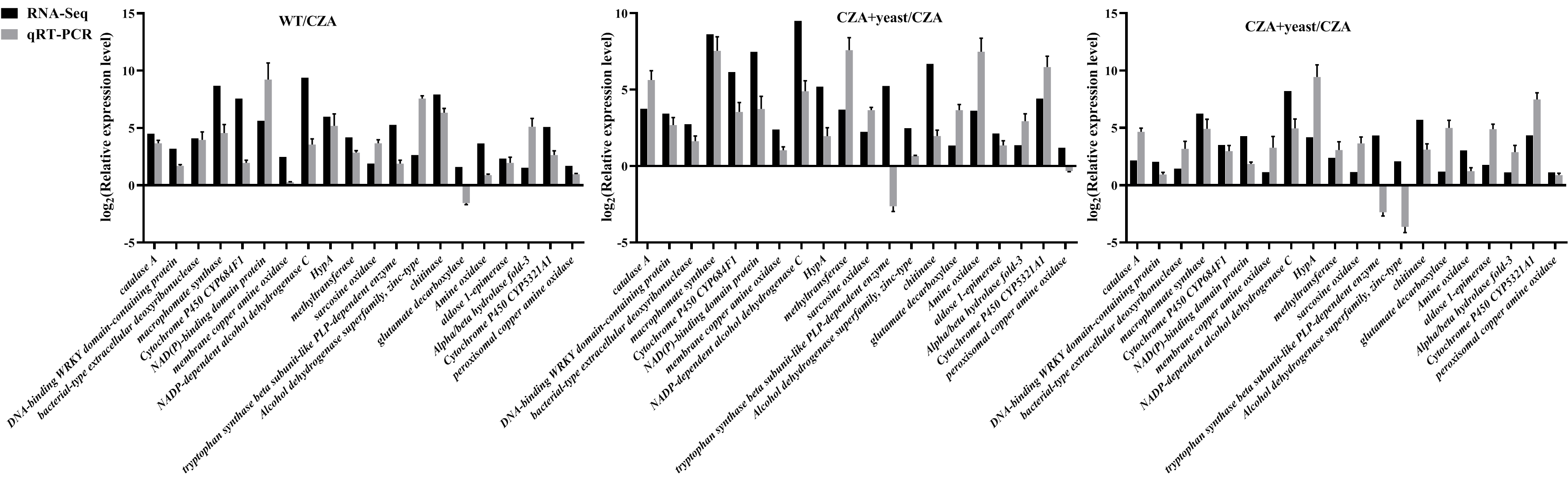

Supplement: Supplementary file 1 [file jof-07-00737-s001.zip › Figure S2.tif]

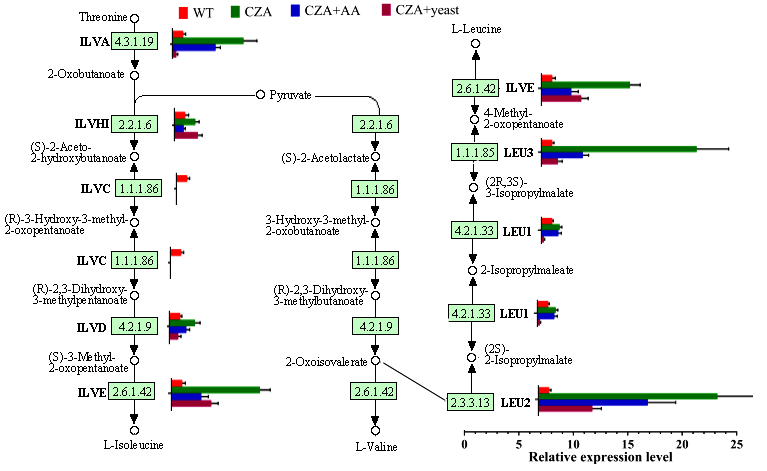

Supplement: Supplementary file 1 [file jof-07-00737-s001.zip › Figure S1.tif]
